# Fermentative Production of the Diamine Putrescine: System Metabolic Engineering of *Corynebacterium Glutamicum*

**DOI:** 10.3390/metabo5020211

**Published:** 2015-04-24

**Authors:** Anh Q. D. Nguyen, Jens Schneider, Gajendar Komati Reddy, Volker F. Wendisch

**Affiliations:** Chair of Genetics of Prokaryotes, Faculty of Biology & CeBiTec, Bielefeld University, Universitätsstr. 25, 33615 Bielefeld, Germany; E-Mails: anguyen@cebitec.uni-bielefeld.de (A.Q.D.N.); jens.schneider@evonik.com (J.S.); gkomati@CeBiTec.Uni-Bielefeld.de (G.K.R.)

**Keywords:** diamine production, putrescine, *Corynebacterium glutamicum*, 2-oxoglutatarate dehydrogenase, pyruvate carboxylase, glyceraldehyde 3-phosphate dehydrogenase, OdhI, spermidine N-acetyltransferase, CgmR, N-acetylglutamate kinase, gamma-glutamate kinase, genome-scale metabolic model, flux balance analysis

## Abstract

*Corynebacterium glutamicum* shows great potential for the production of the glutamate-derived diamine putrescine, a monomeric compound of polyamides. A genome-scale stoichiometric model of a *C. glutamicum* strain with reduced ornithine transcarbamoylase activity, derepressed arginine biosynthesis, and an anabolic plasmid-addiction system for heterologous expression of *E. coli* ornithine decarboxylase gene *speC* was investigated by flux balance analysis with respect to its putrescine production potential. Based on these simulations, enhancing glycolysis and anaplerosis by plasmid-borne overexpression of the genes for glyceraldehyde 3-phosphate dehydrogenase and pyruvate carboxylase as well as reducing 2-oxoglutarate dehydrogenase activity were chosen as targets for metabolic engineering. Changing the translational start codon of the chromosomal gene for 2-oxoglutarate dehydrogenase subunit E1o to the less preferred TTG and changing threonine 15 of OdhI to alanine reduced 2-oxoglutarate dehydrogenase activity about five fold and improved putrescine titers by 28%. Additional engineering steps improved further putrescine production with the largest contributions from preventing the formation of the by-product N-acetylputrescine by deletion of spermi(di)ne N-acetyltransferase gene *snaA* and from overexpression of the gene for a feedback-resistant N-acetylglutamate kinase variant. The resulting *C. glutamicum* strain NA6 obtained by systems metabolic engineering accumulated two fold more putrescine than the base strain, *i.e*., 58.1 ± 0.2 mM, and showed a specific productivity of 0.045 g·g^−1^·h^−1^ and a yield on glucose of 0.26 g·g^−1^.

## 1. Introduction

In 2012, the total world market for polyamides was US $22 billion, and with the increasing growth rate annually, it is expected to reach a market value of US $27 billion by 2018 [1]. Due to their extreme durability and strength, polyamides are used in many applications from textiles, automotives, carpets, sportswear, to oil and gas industry [2,3]. An increasing demand for “green” polyamides is mostly driven by the rising consumer awareness concerning sustainability issues. Polymer precursors can be divided into three major groups: (i) monomers with terminal vinyl groups in polymers such as polystyrene, polyethylene (PE), polyvinylchloride (PVC), *etc.*; (ii) bifunctional monomers with terminal hydroxy, amino, and carboxy functionalities in polymers such as polyesters and polyamides; (iii) diisocyanates in polymers such as polyurethanes. In contrast to isocyanate and vinyl groups, which are rarely compatible with biological systems, the hydroxyl, carboxyl, and amino functional groups of polyamides and polyesters occur throughout the biological world, and their total synthesis by either biocatalysis or fermentation appears feasible [1].

A large number of the polyamide monomers can be produced, in principle, by bio-based routes, which led to the availability of a variety of different polyamides with excellent properties. Successful fermentative production of 1, 4-diaminobutane (putrescine) has recently been demonstrated [4,5]. So far, putrescine has been produced using engineered *E. coli* [6] and *C. glutamicum* [5]. The *C. glutamicum* system was developed further with the highest putrescine yield reported in bacteria 0.26 g·g^−1^ [4]. Tuning expression of ornithine transcarbamoylase gene *argF* over 1000 fold through modulation of transcription and translation efficiencies was the key to balance low-level ornithine transcarbamoylase activity for obtaining high productivity and titer [4]. Putrescine was also produced from alternative carbon sources such as crude glycerol [7], hemicellulosic hydrolysates [8], amino sugars [9], thick juice [10] and by a biotin-prototrophic putrescine producing strain [11].

In this study, a genome-scale stoichiometric model of *C. glutamicum* was investigated by flux balance analysis with respect to the metabolic potential for putrescine production. Subsequently, putrescine production was optimized by engineering glycolysis, anaplerosis, 2-oxoglutarate dehydrogenase activity, proline biosynthesis, putrescine N-acetylation and feedback control of arginine biosynthesis.

## 2. Results and Discussion

### 2.1. In Silico Characterization of Putrescine Production in C. glutamicum

The optimum yield on glucose for *C. glutamicum* is 94% (mol·mol^−1^) and, is 0.627 mol-C·mol-C^−1^ as calculated by flux balance analysis regardless of biomass formation (Table 1). Simulation results suggested that putrescine biosynthesis is constrained by the stoichiometry and furthermore by redox availability, indicated by a positive shadow price for NADH (not shown). Putrescine biosynthesis from carbon sources like lactate and acetate yielded less putrescine, but in contrast to glucose, the production was only constrained by stoichiometry. The putrescine yield on a more reduced carbon source like glycerol was higher (0.653 mol-C·mol-C^−1^) compared to glucose with the synthesis no longer being redox-constrained.

**Table 1:** Theoretical metabolic capacity of *C. glutamicum* for putrescine production with respect to different carbon sources. The carbon uptake was constrained to 24 mmol-C gDCW^−1^·h^−1^. The degree of reduction (Κ) and the yield of putrescine on the indicated carbon source Y_P/S_ are given.

Carbon Source	Κ	Y_P/S_ [mol-C·mol-C^−^^1^]
Glucose	4.0	0.627
Glycerol	4.7	0.653
Lactate	4.0	0.511
Acetate	4.0	0.464

**Figure 1:**
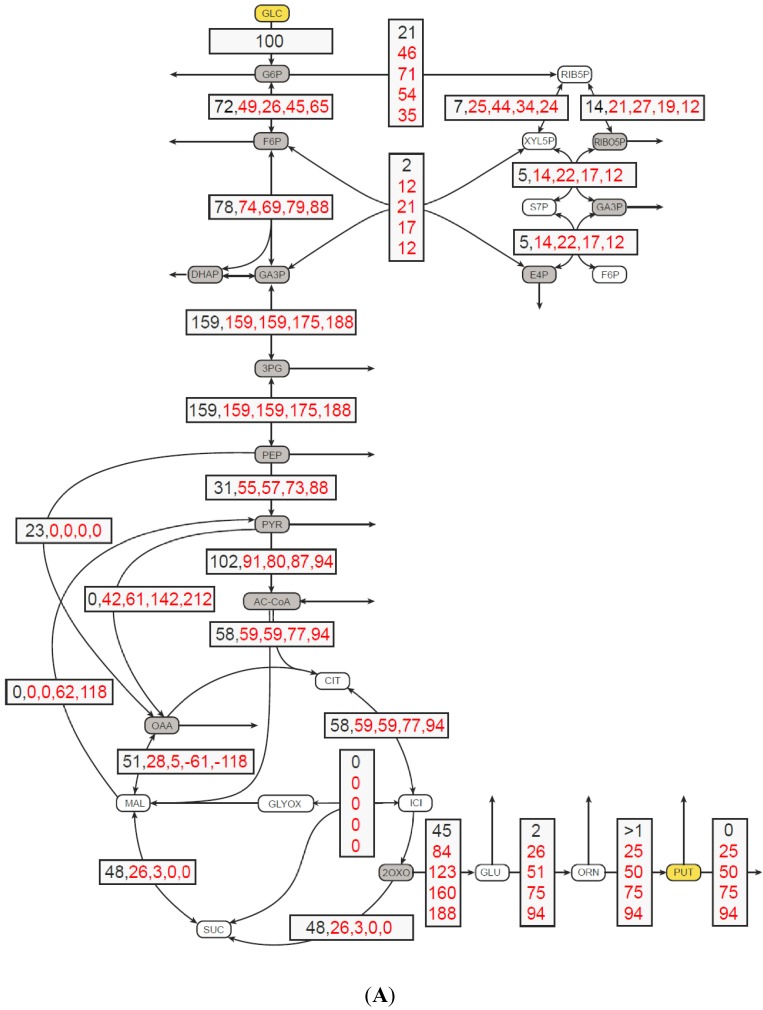

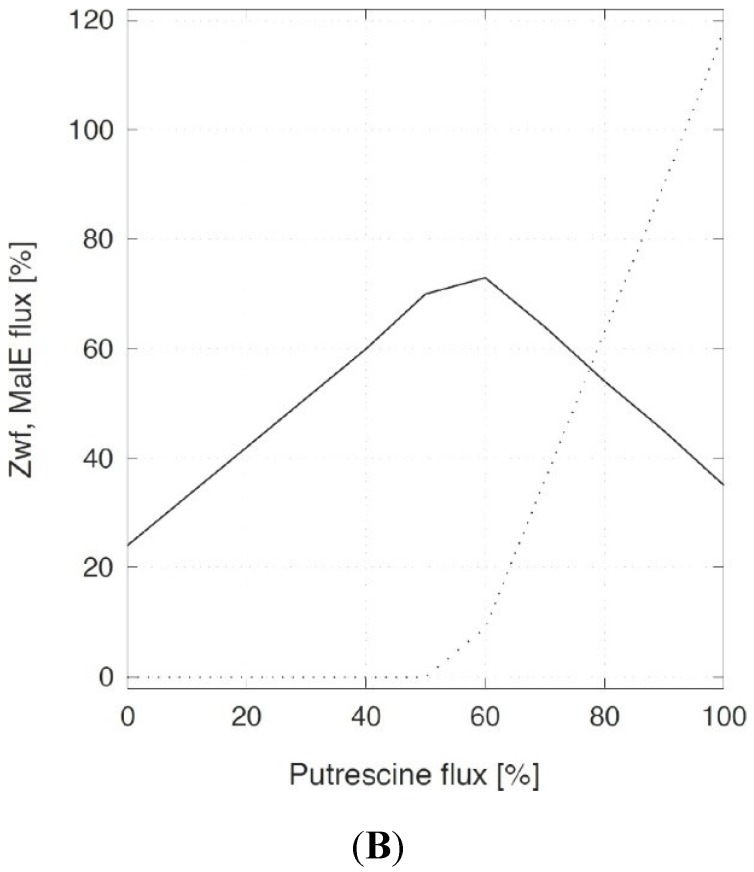
Metabolic flux distribution in *C. glutamicum* (**A**) and the relative flux through glucose 6-phosphate dehydrogenase Zwf and malate enzyme (MalE) (**B**) as a function of putrescine production. (**A**) Objective function was biomass flux, except for 100% putrescine flux. The metabolic flux was distributions were calculated in *C. glutamicum* without (in black) and with (in red) putrescine secretion to obtain yield coefficient (Y_P/S_) of 25, 50, 75, 94%, respectively, relative to the glucose uptake rate. All fluxes are given in percent and are normalized to glucose uptake. Values are sorted by increasing putrescine flux. Solid line: Zwf flux, dotted line: MalE flux. For abbreviations: 1,3PG: 1,3-Bisphosphogylceric acid, 2OXO: 2-Oxoglutaric acid , 2PG: 2-Phosphoglyceric acid, 3PG: 3-Phosphoglyceric acid, AC-CoA: Acetyl-CoA, CIT: Citric acid, DHAP: Dihydroxyacetonephosphate, F6P: Fructose-6-phosphate, G6P: Glucose-6-phosphate, GA3P: Glyceraldyehyde-3-phosphate, GLC: Glucose, GLC-LAC: 6-Phosphogluconolactone, GLC6P: 6-Phosphogluconic acid, GLU: l-Glutamic acid, GLY: Glycerol, GLY3P: Glycerol-3-phosphate, ICI: Isocitric acid, l-RIB5P: l-Ribulose-5-phosphate, MAL: Malic acid, NAC-GLU: N-Acteylglutamic acid, OAA: Oxalacetic acid, ORN: l-Ornithine, PEP: Phosphoenolpyruvic acid, PUT: Putrescine, PYR: Pyruvic acid, RIB: LRibulose, RIB5P: Ribulose-5-phosphate, RIBO5P: Ribose-5-phosphate, S7P: Sedoheptulose-7-phosphate, E4P: Erythrose-4-phosphate, SUC: Succinic acid. Arrows from intermediates marked in grey boxes perpendicular to the metabolic reactions indicate flux into biomass.

The next step was to investigate the *in silico* flux distributions associated with different putrescine production rates (Figure 1). As shown in Figure 1A, the split ratio of carbon flux at the glucose-6-phosphate node (Pgi/Zwf) without putrescine production was 72% to 21%. This ratio differed slightly from the flux measured in the wild type and the flux calculated in simulation experiments by Shinfuku and colleagues, who determined ratios of 59% to 41% and 60% to 40%, respectively [12]. When putrescine secretion had been increased stepwise up to 94%, a flux redistribution was observed. The flux through the pentose phosphate pathway (PPP) increased up to 71% at 50% putrescine production with respect to the glucose uptake, indicating an increased NADPH demand for putrescine production (Figure 1B). Interestingly, if putrescine production increased even further, this did not lead to an increased flux through the PPP. Rather a decrease to 35% was observed at 94% putrescine flux. This decrease was compensated by an active malate enzyme (MalE) at a putrescine flux above 50% (Figure 1B). MalE in combination with pyruvate carboxylase (Pyc) and malate dehydrogenase (Mdh) constitutes a transhydrogenase cycle [13], might supply NADPH for the reduction of glutamate to putrescine.

For biosynthesis of one mole putrescine from glucose three moles NADPH are consumed with one mole NADPH being formed by conversion of glucose to 2-oxoglutarate and three moles of NADPH being consumed by NADP-dependent glutamate dehydrogenase Gdh and NADP-dependent N-acetyl-γ-glutamyl-phosphate reductase ArgC and indirectly by glutamate-dependent N-acetyl-ornithine aminotransferase ArgD. Redox cofactor supply was not engineered in this work, but might prove relevant in further improving putrescine production by recombinant *C. glutamicum*. This may be particularly important when using less reduced carbon sources such as glycerol [7,14], d-gluconate, d-glucuronic acid (a component of plant xylans) or d-galacturonic acid (abundant in pectin-rich waste such as peels and pulps) [15]. Besides redox cofactor regeneration, carbon redirection from the tricarboxylic acid cycle (TCA) towards glutamate biosynthesis is crucial for high putrescine yields. The redirection may be achieved by reducing the 2-oxoglutarate dehydrogenase (OdhA) activity. The flux through 2-oxoglutarate dehydrogenase complex (ODHC) was 48% of wild-type conditions, when flux simulation was done with optimization towards growth. This flux became zero at putrescine yields of 0.75 mol·mol^-1^ or higher.

**Figure 2:**
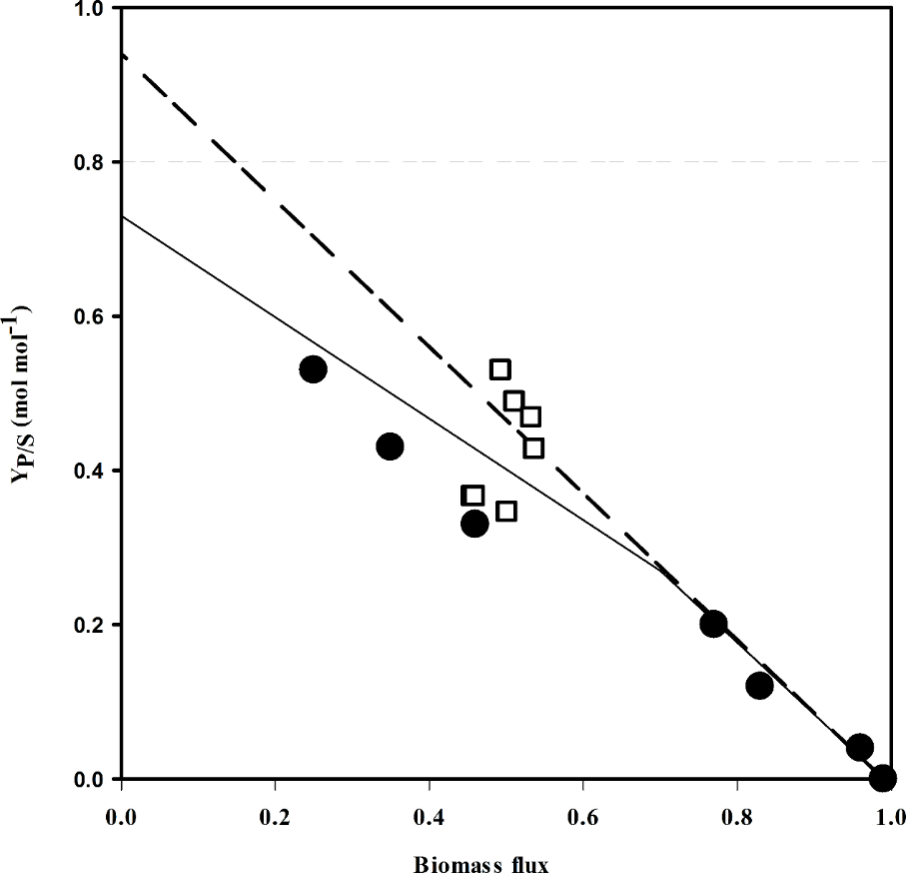
Comparison of theoretical and experimental putrescine yields. The putrescine flux response was analyzed by flux balance analysis with different biomass (the split ratio between Embden-Meyerhof-Parnas pathway (EMP) and pentose phosphate pathway (PPP) pathway was 6:4) when MalE was inactive (solid line) and active (dashed line). Circles: PUT3-27 [4], open squares: NA2-8.

Next, the yields of the current producer strains [4] were compared with those predicted by flux balance analysis. The experimental yields matched the linear increase up to 0.21 mol·mol^-1^ (Table S1, Figure 2). The yield coefficients of the high yielding strains PUT21, PUT24 and PUT27 [4] were marginally lower than predicted for the network structure with constrained PPP and inactive MalE. Taken together, the simulations indicated that cofactor supply, carbon redirection or a combination of both might be limiting the putrescine yield in the current producer strains PUT21, PUT24 and PUT27.

### 2.2. Reducing 2-Oxoglutarate Dehydrogenase Activity as a Target to Increase Putrescine Production

**Figure 3:**
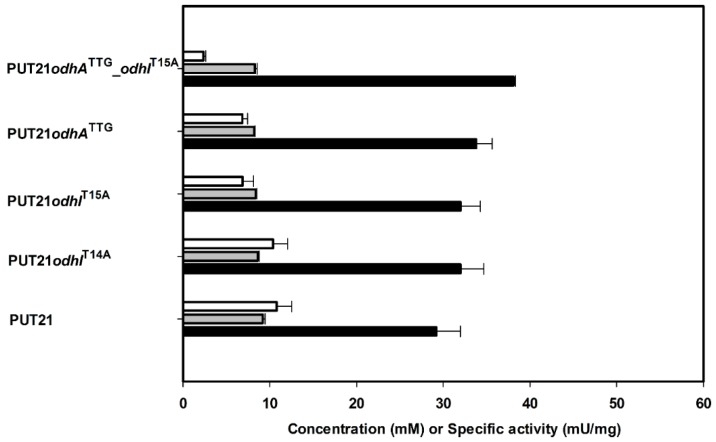
2-Oxoglutarate dehydrogenase as a target to increase production of putrescine production. Concentrations of putrescine (black bar) and N-acetylputrescine (grey bar) in supernatants and 2-oxoglutarate dehydrogenase activities in crude extracts (white bar) of different strains are given as means and standard errors of three independent cultivations. Cells were grown in CGXII medium with 20 g·L^−1^ glucose and 1 mM IPTG.

2-Oxogluatarate is a branch point metabolite, which can be funneled either into the TCA cycle or glutamate biosynthesis. ODHC is a key enzyme in the TCA cycle consisting of three subunits: E1o is encoded by *odhA* [16], E2o is encoded by *aceF*, and E3 is encoded by *lpd* [17]. Putrescine production should benefit from the reduction of ODHC activity as suggested by the flux simulation and two approaches were followed: decreasing synthesis of OdhA, a subunit of ODHC [16] and/or inhibiting ODHC by maintaining its binding with OdhI [18]. Reducing synthesis of OdhA by exchanging the translational start codon from GTG to TTG in the chromosome of strain PUT21 reduced ODHC activity from 11 ± 2 mU/mg to 7 ± 1 mU/mg (Figure 3) and increased putrescine production by 15% (Figure 3). Alternatively, maintaining inhibition of ODHC by exchanging threonine residues 14 or 15 or both of OdhI to alanine resulted in partial or complete loss of phosphorylation and inactivation of OdhI by PknG [18]. Strains PUT21*odhI*^T14A^ and PUT21*odhI*^T15A^ showed ODHC activities of 10 ± 2 and 7 ± 1 mU/mg, respectively (Figure 3) and produced 32 ± 3 mM and 32 ± 2 mM putrescine (Figure 3). Combining the chromosomal changes *odhA*^TTG^ and *odhI*^T15A^ yielded strain NA2, reduced ODHC activity to 2 ± 0 mU/mg (Figure 3) and increased putrescine production by 28% (38.1 ± 0.2 mM) as compared to PUT21 (29.2 ± 2.8 mM, Figure 3). Significant concentrations (>2 mM) of glutamate or other amino acids were not detected in culture broth at the end of all cultivations.

**Table 2:** Growth Rates and Putrescine Production Parameters Obtained with Various Engineered *C. glutamicum* Strains ^a^.

Strain	Growth Rate µ^b^ (h^−1^)	Putrescine Accumulation (mM)	Product Yield Y_P/S_^b^ (g·g^−1^)	Biomass Yield Y_X/S_^b^ (g·g^−1^)	Volumetric Productivity Q_p_^c^ (g·L^−1^·h^−1^)	Specific Productivity q_p_^c^ (g·g^−1^·h^−1^)
PUT21	0.19	29.2 ± 2.8	0.13	0.28	0.11	0.020
PUT21*proB*^GTG^	0.22	31.2 ± 3.0	0.14	0.27	0.12	0.022
PUT21*proB*^TTG^	0.14	46.1 ± 2.8	0.20	0.29	0.14	0.024
NA2	0.17	38.1 ± 0.2	0.17	0.25	0.14	0.028
NA3	0.16	41.9 ± 1.1	0.18	0.22	0.14	0.032
NA4	0.16	41.6 ± 1.1	0.18	0.26	0.14	0.027
NA5	0.15	48.3 ± 3.3	0.21	0.23	0.19	0.041
NA6	0.17	58.1 ± 0.2	0.26	0.23	0.21	0.045
NA7	0.20	53.4 ± 2.2	0.24	0.24	0.20	0.042
NA8	0.20	51.2 ± 0.5	0.23	0.24	0.20	0.041

^a^ Means of three independent cultivations in CGXII medium with 20 g L^-1^ glucose and 1 mM IPTG are given. ^b^ Relative standard errors were 10% or less. ^c^ Relative standard errors were 15% or less.

Low or even absent ODHC activity has been associated with glutamate overproduction by *C. glutamicum* since long (see, e.g., [19]) and deletion of *odhA* led to overproduction of glutamate [20]. The importance of ODHC in *C. glutamicum* metabolism is reflected in its elegant activity control [21]. In its unphosphorylated form, the small protein OdhI inhibits ODHC activity [18] by direct interaction of its FHA domain with the E1o subunit of ODHC [22]. The protein kinases PknG, PknA, PknB and PknL phosphorylate OdhI at T14 and/or T15 [18,23,24]. Manipulation of OdhI phosphorylation by mutation of the threonine residues inOdhI or by deletion of the gene for protein kinase PknG has been shown previously to increase glutamate production [24]. In the present study, reducing translation of *odhA* due to changing the translational start codon GTG to the less preferred TTG and exchanging the threonine residue 15 of OdhI, which is phosphorylated by the protein kinases, were combined. As consequence, about a five fold reduced ODHC activity was observed (Figure 3), but growth hardly slowed (compare NA2 with PUT21 in Table 2) (growth rate of 0.16 h^−1^). This may explain why indications of genetic instability were not observed with the strains described here, whereas several different classes of suppressor mutants arose upon deletion of *odhA* [20] and, thus, reducing ODHC activity may be superior to its complete absence.

### 2.3. Increasing Precursor Supply Flux for Putrescine Production

**Figure 4:**
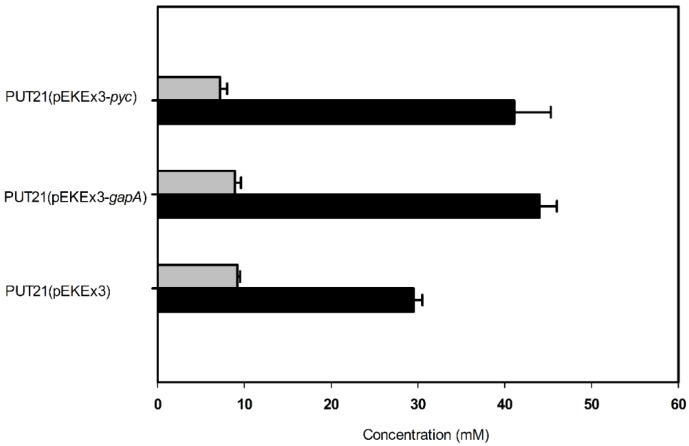
Effect of pEKEx3-based overexpression of *gapA* and *pyc* in PUT21 on the production of putrescine (black bar) and N-acetylputrescine byproduct (grey bar). Cells were grown in CGXII medium with 20 g·L^−1^ glucose and 1 mM isopropyl β-d-1-thiogalactopyranoside (IPTG). Means and standard error of three independent cultivations are shown.

In order to increase the supply of 2-oxoglutarate as precursor for putrescine biosynthesis, two targets were chosen: glyceraldehyde 3-phosphate dehydrogenase in glycolysis and pyruvate carboxylase for anaplerosis of the TCA cycle. Since glyceraldehyde 3-phosphate dehydrogenase has been shown to be limiting glycolytic flux at least under oxygen-deprivation conditions [25], overexpression of its gene *gapA* in PUT21 was tested. As compared to PUT21 carrying the empty vector, PUT21 overexpressing *gapA* produced 39% more putrescine (44.0 ± 2.0 mM as compared to 29.5 ± 1.0 mM, Figure 4).

Since pyruvate carboxylase is the major anaplerotic enzyme in *C. glutamicum* [26] and overexpression of its gene improved glutamate and lysine production [27], *pyc* was overexpressed in PUT21. *C. glutamicum* PUT21(pEKEx3-*pyc*) produced 41 ± 4 mM putrescine, an increase by 39% as compared to the empty vector carrying control strain (29 ± 1 mM, Figure 4).

Overexpression of *pyc* and *gapA* improved putrescine accumulation (Figure 4), but the individual effects were not additive (compare NA3 and NA4 in Table 2 with Figure 4). Whereas overexpression of *pyc* was expected to increase anaplerosis and putrescine yield as reported previously for lysine and glutamate production [27], overexpression of *gapA* was expected to accelerate glucose utilization [28] and in consequence productivity. The simulation only showed a small flux increase from glyceraldehyde 3-phosphate to 3-phosphoglycerate in response to an increased theoretical putrescine yield, which is consistent with metabolic flux analysis data reported previously for glutamate overproduction in *C. glutamicum* [29]. Overexpression of *gapA* from plasmid pVWEx1 did neither change the growth rate nor the volumetric productivity, but improved the specific productivity due to reduced biomass formation (compare NA2 and NA3 in Table 2). It is not clear if *gapA* overexpression led to faster glucose consumption. The positive effect of *gapA* overexpression on putrescine accumulation varied with the expression plasmid used. Based on plasmid pVWEx1, GAPDH activity increased to 160 ± 10 mU/mg, while plasmid pEKEx3 led 260 ± 10 mU/mg [30]. This might explain the relatively small effect observed in *C. glutamicum* NA3.

### 2.4. Decreasing Conversion of Glutamate to Proline

**Figure 5:**
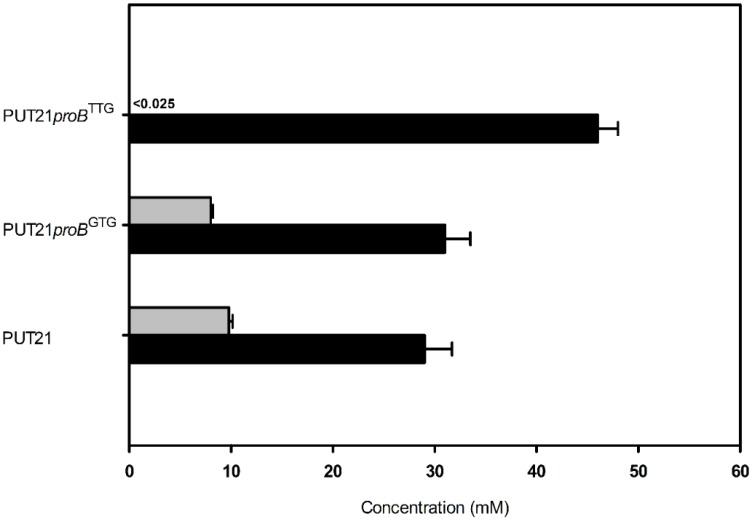
Putrescine production (black bar) and N-acetylputrescine byproduct (grey bar) in PUT21-derived strains carrying *proB* with different translational start codons. Cells were grown in CGXII medium with 20 g·L^−1^ glucose and plasmid encoded genes were induced with 1 mM IPTG. Means and standard error of three independent experiments are shown.

As putrescine production competes with proline biosynthesis for glutamate, it was tested if reducing proline biosynthesis positively affects putrescine production. To this end, the translational start codon of *proB* encoding γ-glutamate kinase, the initial enzyme of proline biosynthesis, was exchanged from ATG to GTG and TTG in the chromosome of strain *C. glutamicum* PUT21 by replacement mutagenesis. Indeed, putrescine production increased when the *proB* gene contained the less preferred translational start codons GTG and TTG (Figure 5). Notably, PUT21*proB*^TTG^ did not produce N-acetylputrescine as a side product and 46.1 ± 2.8 mM putrescine accumulated. Thus, putrescine production benefitted from reducing entry of glutamate into proline biosynthesis.

Similarly, deletion of *proB* has been shown to improve ornithine production [31,32], however, as a consequence the resulting strains require either complex media components or addition of proline as a supplement to the culture medium. Interestingly, strain PUT21*proB*^TTG^ produced more putrescine, but no N-acetylputrescine accumulated (Figure 5), although *snaA*, the gene for spermi(di)ne N-acetyltransferase, was intact. This effect is currently not understood, but activity or synthesis of spermi(di)ne N-acetyltransferase may be reduced in PUT21*proB*^TTG^ due to higher levels of glutamate or lower levels of proline or intermediates of proline biosynthesis. Proline is known to affect binding of ArgR to the *argB* promoter region [33], but this does not pertain to the putrescine strains described here because *argR* is deleted in all of them. Proline is the major compatible solute of *C. glutamicum* and is produced in response to high osmolality [34] or taken up into the cell by osmo-regulated EctP and ProP or for anabolic purposes by PutP [35]. It is currently unknown if *snaA* expression or SnaA activity is osmo-regulated. Nonetheless, the effect of *proB*^TTG^ on abrogating N-acetylputrescine as a side product improved putrescine titers.

### 2.5. Combinatorial Construction of Putrescine Overproducing C. glutamicum Strains

**Figure 6:**
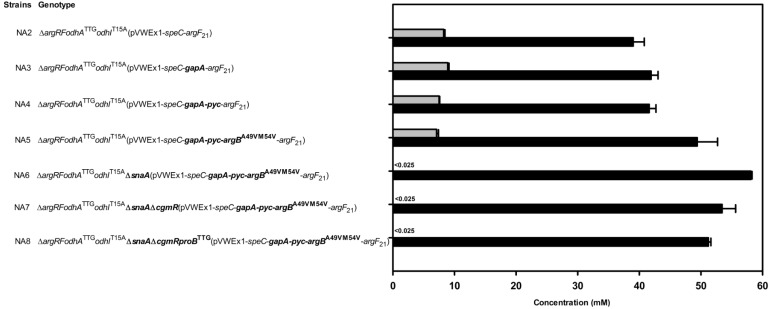
Effect of deletion and overexpression of engineering target genes in *C. glutamicum* strain NA2 on the production of putrescine (black bars) and N-acetylputrescine (grey bars). Genetic changes introduced to the chromosome of *C. glutamicum* NA2 and to plasmid pVWEx1-*speC*-*argF*_21_ are highlighted in bold. Genes for feedback-resistant N-acetylglutamate kinase (*argB*^A49V/M54V^), pyruvate carboxylase (*pyc*) and glyceraldehyde 3-phosphate dehydrogenase (*gapA*) were added to plasmid pVWEX1-*speC-argF*_21_, the translational start codon exchange of the γ-glutamate kinase gene *proB* from ATG to TTG was introduced in the chromosome, while the spermi(di)ne N-acetyltransferase gene *snaA* and the regulatory gene *cgmR* were deleted. Cells were grown in CGXII medium with 20 g·L^-1^ glucose and 1 mM IPTG. Means and standard errors of three independent cultivations are shown.

Strain improvement was based on *C. glutamicum* PUT21, which produced 29.2 ± 2.8 mM putrescine from 20 g·L^−1^ glucose (Table 2). Strain NA2 carried the chromosomal changes *odhA*^TTG^ and *odhI*^T15A^ and produced 38.1 ± 0.2 mM (Table 2). Overexpression of *gapA* in NA2 yielded strain NA3, which showed increased glyceraldehyde 3-phosphate dehydrogenase activity (160 ± 10 as compared 130 ± 10 mU/mg) and produced 41.9 ± 1.1 mM putrescine (Table 2, Figure 6). Additional overexpression of *pyc* did not increase putrescine production by strain NA4 (Figure 6). However, NA5, which showed increased feedback-resistant N-acetyl glutamate kinase activity due to additional overexpression of a*rgB*^A49V/M54V^ [36], produced more putrescine, namely 48.3 ± 3.3 mM (Figure 6). Overexpression of *argB*^A49V/M54V^ improved putrescine overproduction although arginine accumulation was avoided in all putrescine producing strains described in this study due to leaky expression of *argF*. Thus, it is possible that overexpression of genes for various other feedback-alleviated N-acetylglutamate kinase variants [37,38] may increase putrescine production further. Although all N-acetylglutamate kinase variants have been selected for loss of feedback inhibition by arginine, it is conceivable that N-acetylglutamate kinase is inhibited by putrescine, ornithine or other arginine biosynthesis intermediates and that some of the described variants may have lost this putative feedback inhibition as well.

Recently, N-acetylation of putrescine was identified and the responsible protein SnaA characterized as spermi(di)ne N-acetyltransferase [39]. Deletion of *snaA* in *C. glutamicum* NA5 abrogated formation of N-acetylputrescine as a side-product and 58.1 ± 0.2 mM putrescine accumulated (Figure 6). Since the TetR-family transcriptional repressor CgmR represses *cgmAR* expression in dependence of diamines [39,40] and since overexpression of the putative export permease gene *cgmA* improved putrescine and cadaverine production [39,41], the repressor gene *cgmR* was deleted in *C. glutamicum* NA6 to yield strain NA7. However, putrescine production by strain NA7 was not improved (53.4 ± 2.2, Table 2), but slightly reduced as compared to strain NA6 (Figure 6). The translational start codon exchange from ATG to TTG of the chromosomal proline biosynthesis gene *proB* in *C. glutamicum* NA7 yielded strain NA8, which produced less putrescine (51.2 ± 0.5 mM) than strain *C. glutamicum* NA6 (Figure 6).

Deletion of *snaA* was beneficial for improving putrescine production by abrogating formation of N-acetylputrescine as by-product (Figure 6) as observed previously. Since all strains lacking SnaA (NA6, NA7, NA8 in Figure 6 and strains described in [39]) did not accumulate N-acetylputrescine, other N-acetyltransferases including ornithine N-acetyltransferase ArgJ, which is overexpressed in the putrescine producing strains, did not compensate for the lack of SnaA. SnaA acetylates a number of diamines and triamines including cadaverine using acetyl-CoA or propionyl-CoA as donors [39] and deletion of *snaA* also improved production of the diamine cadaverine by abrogating formation of N-acetylcadaverine as by-product [42]. Overexpression of putative putrescine permease gene *cgmA* or deletion of *cgmAR* repressor CgmR improved putrescine formation [39], but not in a Δ*snaA* background as observed here (compare NA6 and NA7 in Figure 6) and previously [39]. However, it is not known if N-acetylputrescine affects CgmA or CgmR or if the higher putrescine concentrations in strains lacking SnaA are sufficient to trigger maximal putrescine export.

Production results from strains NA2 to NA8 were compared with data predicted by flux balance analysis (Table S1, Figure 2). The yield coefficients of strains NA2, NA3 and NA4 were slightly lower than predicted with constrained PPP and inactive MalE. However, data obtained for strains NA5 to NA8 fitted the prediction when MalE is active. Further analysis needs to be done to confirm the role of MalE in these strains.

Taken together, combinatorial strain development led to the efficient putrescine producer *C. glutamicum* NA6, which accumulated 58.1 ± 0.2 mM putrescine with a yield on glucose of 0.26 g·g^−1^ and a volumetric productivity of 0.21 g·L^−1^·h^−1^. As shown previously, product titers can be increased in fed-batch processes, e.g., up to 211 mM [4]. Strain NA6 showed the same product yield as PUT24 [4], however, the maximal putrescine titer was reached twice faster. The highest specific productivity of 0.75 g·L^−1^·h^−1^ has been described for *E. coli* in a fed-batch process [6]. Since this *E. coli* strain showed a yield of 0.168 g·g^−1^ [6], while *C. glutamicum* NA6 showed a yield of 0.26 g·g^−1^, it appears possible to achieve similar or even higher specific productivities than with the *E. coli* strain.

These putrescine production capability was due to improved precursor supply (overexpression of *gapA* and *pyc*; reduced ODHC activity as consequence of *odhA*^TTG^ and *odhI*^T15A^, reduced ornithine transcarbamoylase activity due plasmid-borne expression of *argF*_21_ and chromosomal *argF* deletion), abrogated N-acetylation of putrescine (*snaA* deletion), derepressed arginine biosynthesis (*argR* deletion) and feedback-resistant N-acetylglutamate kinase (a*rgB*^A49V/M54V^), and heterologous expression of *E. coli* ornithine decarboxylase gene *speC* via an anabolic plasmid-addiction system.

## 3. Experimental Section

### 3.1. Bacterial Strains and Culture Conditions

Strains and plasmids used in the present work are listed in Table 3. *C. glutamicum* and *E. coli* strains were routinely grown in lysogeny broth (LB) (10 g·L^−1^ tryptone, 5 g·L^−1^ yeast extract, 10 g·L^−1^ sodium chloride) in 500 mL baffled flasks on a rotary shaker (120 rpm) at 30 °C or 37 °C. Briefly, a 50 mL LB seed culture of *C. glutamicum* was inoculated from an agar plate and grown overnight. The cells were harvested by centrifugation (4000 × g, 10 min) and washed once with CGXII minimal medium [43] lacking the carbon source. Subsequently, 50 mL CGXII medium, containing 20 g·L^−1^ glucose and necessary supplements, was inoculated to an initial optical density at 600 nm of 0.5. Growth was followed by measuring the optical density at 600 nm. The biomass concentration was calculated from OD_600_ values using an experimentally determined correlation factor of 0.25 g cell dry weight (DW) L^−1^ for OD_600_ of 1. When necessary, the growth medium was supplemented with kanamycin (25 μg·mL^−1^), spectinomycin (100 μg·mL^−1^), and isopropyl β-d-1-thiogalactopyranoside (IPTG) (1 mM).

**Table 3:** *C. glutamicum* Strains and Plasmids.

Name	Relevant Genotype/Information	Refs.
Strains		
**ORN1**	In-frame deletion of *argR* and *argF*, l-ornithine overproducing strain derived from *C. glutamicum* ATCC13032; auxotrophic for l-arginine	[5]
**PUT21**	ORN1 carrying plasmid pVWEx1-*speC*-*argF*_21_	[4]
**PUT21*odhA*^TTG^**	PUT21 with replacement of translational start codon GTG of chromosomal *odhA* of by TTG	This study
**PUT21*odhI*^T14A^**	PUT21 with replacement of threonine codon 14 of *odhI* by an alanine codon	This study
**PUT21*odhI*^T15A^**	PUT21 with replacement of threonine codon 15 of chromosomal *odhI* by an alanine codon	This study
**PUT21*proB*^GTG^**	PUT21 with replacement of translational start codon ATG of chromosomal *proB* by GTG	This study
**PUT21*proB*^TTG^**	PUT21 with replacement of translational start codon ATG of chromosomal *proB* by TTG	This study
**NA2**	PUT21*odhA*^TTG^ with replacement of threonine codon 15 of chromosomal *odhI* by an alanine codon	This study
**NA3**	NA2, but carrying plasmid pVWEx1-*speC*-*gapA*-*argF*_21_ instead of pVWEx1-*speC*-*argF*_21_	This study
**NA4**	NA2, but carrying plasmid pVWEx1-*speC*-*gapA*-*pyc*-*argF*_21_ instead of pVWEx1-*speC*-*argF*_21_	This study
**NA5**	NA2, but carrying plasmid pVWEx1-*speC*-*gapA*-*pyc*-*argB^A49V/M54V^*-*argF*_21_ instead of pVWEx1-*speC*-*argF*_21_	This study
**NA6**	NA5 with chromosomal deletion of *snaA*	This study
**NA7**	NA6 with chromosomal deletion of *cgmR*	This study
**NA8**	NA7 with replacement of translational start codon ATG of chromosomal *proB* by TTG	This study
Plasmids		
pEKEx3	Spec^R^; Ptac, lacIq; pBL1, oriVC.g., oriVE.c.	[44]
pEKEx3-*gapA*	SpeC^R^, pEKEx3 overexpressing *gapA* from *C. glutamicum* ATCC13032	[30]
pEKEx3-*pyc*	SpeC^R^, pEKEx3 overexpressing *pyc* from *C. glutamicum* ATCC13032	[27]
pEKEx3-*argB^A49V/M54V^*	SpeC^R^, pEKEx3 overexpressing *argB^A49V/M54V^* from *C. glutamicum* ATCC13032	[36]
pVWEx1-*speC*-*argF*_21_	Kan^R^ , plasmid-based overexpressing *speC* from *E. coli* MG1655 and leaky expression of *argF* in pVWEx1	[4]
pK19*mobsacBodhA*^TTG^	Kan^R^; mobilizable vector for the replacement of start codon of *odhA* from GTG to TTG	This study
pK19*mobsacBodhI*^T14A^	Kan^R^; mobilizable vector for the replacement of threonine 14 in *odhI* by alanine	This study
**pVWEx1-*speC*-*gapA-pyc-argB*^A49V/M54V^-*argF*_21_**	Kan^R^, Kan^R^, plasmid-based overexpressing *argB*^A49V/M54V^, *pyc* and *gapA* from *C. glutamicum,* *speC* from *E. coli* MG1655 and leaky expression of *argF*	This study
**pK19*mobsacB***	Kan^R^; mobilizable *E. coli* vector for the construction of deletion mutants in *C. glutamicum* (oriVE.c. , PT7, lacI)	[45]
**pK19*mobsacBodhA*^TTG^**	Kan^R^; mobilizable vector for the replacement of start codon of *odhA* from GTG to TTG	This study
**pK19*mobsacBodhI*^T14A^**	Kan^R^; mobilizable vector for the replacement of threonine 14 in *odhI* by alanine	This study
**pK19*mobsacBodhI*^T15A^**	Kan^R^; mobilizable vector for the replacement of threonine 15 in *odhI* by alanine	This study
**pK19*mobsacBproB*^GTG^**	Kan^R^; mobilizable vector for replacement of native start codon ATG of *proB* by GTG	This study
**pK19*mobsacBproB*^TTG^**	Kan^R^; mobilizable vector for replacement of native start codon ATG of *proB* by TTG	This study
**pK19*mobsacB*Δ*snaA***	Kan^R^; mobilizable vector for deletion of *snaA*	[39]
**pK19*mobsacB*Δ*cgmR***	Kan^R^; mobilizable vector for deletion of *cgmR*	[39]

### 3.2. Strain Construction

The oligonucleotides used as PCR primers in this study are listed in Table 4. Plasmids were constructed in *Escherichia coli* DH5α by standard molecular genetic methods and confirmed by DNA sequence analysis. Transformation of *E. coli* was performed using the rubidium chloride method [46] while *C. glutamicum* was transformed by electroporation as described previously [43]. *C. glutamicum* deletion mutants were constructed by crossover PCR (or Gibson Assembly) and double homologous recombination using the suicide vector pK19*mobsacB* [45]. For plasmid-based overexpression, ORFs of corresponding genes were amplified using PCR and ligated into digested plasmid pEKEx3 or pVWEx1 or cloned by Gibson Assembly [47]. The resulting strains are listed in Table 3.

**Table 4:** List of Primers.

Primer names	Sequence (5′-3′)
*odhI*141	CGAATCCATTCACCTGC
*odhI*142	ACTGAGGTGGCCTCGACCTG
*odhI*143	CAGGTCGAGGCCACCTCAGT
*odhI*144	GCAACCGCACTGTTTG
*odhI*152	ACTGAGGCGGTCTCGACCTG
*odhI*153	CAGGTCGAGACCGCCTCAGT
*odhA*1	CCTGATGGTTTCAACCATCAAGTC
*odhA*2	AGTACTAGCGCTGCTCAAGGCAGG
*odhA*3	CTGCCTTGAGCAGCGCTAGTAC
*odhA*4	CCATGGCGTAGCCAATGATG
gtg1	AGCAGTTGGCTACCTGG
gtg2	CACCGGCGCCACTTGGGTTG
gtg3	CAACCCAAGTGGCGCCGGTG
gtg4	GGCAAAAGAACGTCCCC
ttg2	ACCGGCGCCAATTGGGTTGG
ttg3	CCAACCCAATTGGCGCCGGT
*gapA*-cgl-fw	AAGGAGATATAGATATGACCATTCGTGTTGGTATTAAC
*gapA*-cgl-rv	TTAGAGCTTGGAAGCTACGAGCTC
ACBF-*gapA*1	TTGTACGGTTATGTGTTGAAGTAAGGATCCGAAAGGAGGCCCTTCAGATGACCATTCGTGTTGGTATTA
ACBF-*gapA*2	ATCTGAAGGGCCTCCTTTCACATGTTTAGAGCTTGGAAGCTACGAG
ACBF-*gapA*3	AGTGAATTCGAGCTCGGTACCCGGGCATATGTTAGAGCTTGGAAGCTACGAG
ACBF-*pyc*1	CTCGTAGCTTCCAAGCTCTAAACATGTGAAAGGAGGCCCTTCAGATGTCGACTCACACATCTTC
ACBF-*pyc*2	GGCCTCCTTTCGCGGCCGCTTAGGAAACGACGACGATCA
ACBF-*pyc*3	AGTGAATTCGAGCTCGGTACCCGGGCATATGTTAGGAAACGACGACGATCA
ACBF-*argB*1	TGATCGTCGTCGTTTCCTAAGCGGCCGCGAAAGGAGGCCCTTCAGTTG
ACBF-*argB*2	AGTGAATTCGAGCTCGGTACCCGGGCATATGTTACAGTTCCCCATCCTTGTC

### 3.3. Flux Balance Analysis

The flux balance analysis was carried out using Matlab (Mathworks, Natick, MA, USA) and the COBRA 2.0 toolbox, together with the GNU Linear Programming Kit [48]. The genome scale model of *C. glutamicum* was taken from [49]. The following changes were made to the original model: (1) putrescine secretion capability was added, (2) l-arginine interconversion was constrained to zero, (3) glycerol and lactate uptake was added, (4) cofactor of the glycerol-3-phosphate dehydrogenase was changed to NAD, (5) the acetate kinase reaction was set reversible to allow acetate consumption, and (6) NAD(P)H futile reactions were added for capacity estimations (see also supplementary Table S2). All flux units are in mmol (gCDW h)^−1^, except for the flux into biomass formation, which has units of h^−1^. The substrate uptake was varied between 0 and 4 mmol (gCDW h)^−1^. Non growth associated ATP demand was set zero. All other fluxes were left unconstrained, if not otherwise mentioned. Finally, the identification of a particular flux distribution was formulated as a constrained linear programming problem, in which the objective function was maximized. The objective function was (1) biomass formation, (2) putrescine secretion, or (3) cofactor production (ATP, NADH, NADPH). Shadow prices were determined using COBRA.

### 3.4. Enzyme Assays

2-oxoglutarate dehydrogenase activity was assayed as described previously [50]. Only fresh extracts were used, prepared in 0.1 M TES [*N*-tris (hydroxymethyl) methyl-2-aminoethanesulfonic acid] (pH 7.2), 10 mM MgCl_2_, 3 mM cysteine, 10 vol-% glycerol. The assay mixture (1 mL) was 50 mM TES (pH 7.7), 10 mM MgCl_2_, 3 mM cysteine, 2 mM NAD, 0.9 mM thiamine diphosphate, 0.05 mM chlorpromazine, and 2.5 mM 2-oxoglutarate. The reaction was performed at 30 ^o^C, started with 0.2 mM CoA, and extinction followed at 340 nm.

Glyceraldehyde 3-phosphate dehydrogenase activity was measured as previously reported [51]. The assay contained 1 mM NAD^+^, 50 mM Na_2_HPO_4_, 0.2 mM EDTA, and 0.5 mM glyceraldehyde 3-phosphate in 50 mM triethanolamine hydrochloride (TEA) buffer pH 8.5, at 30 ^o^C. One unit of enzyme activity corresponds to 1 μmol NADH formed per minute.

N-Acetylglutamate 5-phosphotransferase activity was performed based on previous report [52]. The incubation mixture consisted of: 400 mM NH_3_OH-HCl, 400 mM Tris (base), 40 mM N-acetyl-l-glutamate, 20 mM MgCl_2_, 10 mM ATP and up to 0.03 unit of enzyme in a final volume of 1.0 mL. The pH of the incubation mixture containing all components except N-acetyl-l-glutamate and enzyme was adjusted to 7.2 with NaOH at 25 °C. The reaction was started by the addition of N-acetyl-l-glutamate. After incubation at 30 °C for 10–20 min the reaction was terminated by the addition of 1.0 mL of a solution containing 5% (w/v) FeCl_3_ hexahydrate, 8% (w/v) trichloroacetic acid, and 0.3 M HCl. When necessary the precipitate was removed by centrifugation. The volume of all reagents was halved for the assay of purified enzyme. The absorbance of the resulting hydroxamate-Fe^3+^ complex was measured at 540 nm. One unit of N-acetylglutamate 5-phosphotransferase is defined as the amount of enzyme which catalyzes the formation of of 1 µmol of product per min under assay conditions.

### 3.5. Quantification of Putrescine

For quantification of extracellular putrescine, aliquots of the culture were withdrawn, the optical density (OD_600_) was measured and cells were removed by centrifugation (13,000 × g, 10 min). The supernatant was analyzed using a high-pressure liquid chromatography system (HPLC, 1200 series, Agilent Technologies Deutschland GmbH, Böblingen, Germany).

Putrescine were determined by precolumn derivatization with ortho-phthaldialdehyde and separated on a reversed phase column (LiChrosphere 125 × 4 mm, 5-μmparticle size, end capped; Chromatographie Service GmbH, Langerwehe, Germany). The respective fluorescent isoindole derivatives were detected by excitation at 330 nm (emission at 450 nm). For elution gradients of 2.5 g·L^−1^ sodium acetate, pH 6.0, as the polar phase and methanol as the nonpolar phase were used. Diaminohexane was used as an internal standard [11].

## 4. Conclusions

Taken together, the previously available putrescine producing strain *C. glutamicum* PUT21 was improved by combinatorial engineering guided by flux balance analysis. In shake flask cultivations 58.1 ± 0.2 mM or about 5.1 g·L^−1^ putrescine accumulated and *C. glutamicum* NA6 produced putrescine with 56% of the maximal product yield (*i.e*., 0.26 g·g^−1^). An overall volumetric productivity of 0.21 g·L^−1^·h^−1^, an overall specific productivity of 0.045 g·g^−1^·h^−1^, and a biomass yield of 0.23 g·g^−1^ were observed (Table 2). Three of the chosen metabolic engineering targets led to the largest improvements: feedback alleviated N-acetylglutamate kinase, reduced ODHC activity and preventing N-acetylation of putrescine.

## Supplementary Files

Supplementary File 1
