# Supplementary material for: Fermentative Production of the Diamine Putrescine: System Metabolic Engineering of Corynebacterium Glutamicum
Source: Metabolites. 2015 Apr 24;5(2):211–31. doi: 10.3390/metabo5020211 (PMC4495370; doi:10.3390/metabo5020211)
Supplement: Supplementary File 1 [file metabolites-05-00211-s001.pdf]

Supplementary Information

Table S1. Production Yield $Y_{P/S}$ and Biomass Flux from Different Putrescine Producing *C. Glutamicum* Strains.

**Strain**	**$Y_{P/S}$ (mol·mol^{-1})**	**Biomass flux (h·h^{-1})**	**Ref.**
PUT3,9,6	0.00	0.99	[1]
PUT12	0.04	0.96	[1]
PUT18	0.12	0.84	[1]
PUT15	0.20	0.77	[1]
PUT21	0.33	0.46	[1]
PUT27	0.43	0.35	[1]
PUT24	0.53	0.25	[1]
NA2	0.35	0.50	This study
NA3	0.37	0.45	This study
NA4	0.37	0.46	This study
NA5	0.43	0.54	This study
NA6	0.53	0.49	This study
NA7	0.49	0.51	This study
NA8	0.47	0.53	This study

Table S2. Changes Made to the Stoichiometric Model of *Corynebacterium Glutamicum* [2].

**Reaction Stoichiometry**
[1] [c]CPD-217 <=> 24DINH2-PENTANOATE
[1] [c]CPD-299 + CO-A <=> D-ALANINE + ACETYL-COA
[1] [c]D-PROLINE + NADH + PROTON <=> 5-AMINOPENTANOATE + NAD
[1] [c]L-ORNITHINE + 2-KETOGLUTARATE <=> GLT + L-GLUTAMATE-GAMMA-SEMIALDEHYDE
[1] [c]L-ORNITHINE <=> CPD-217
[1] [c]NADP + WATER + 24DINH2-PENTANOATE <=> AMMONIA + NADPH + CPD-299 + PROTON
[1] [c]PRO <=> D-PROLINE
[c]ACETYL-P + ADP <=> ACET + ATP + PROTON
[c]GLYCEROL-3P + NAD -> DIHYDROXY-ACETONE-PHOSPHATE + NADH
L-LACTATE[c] <=> L-LACTATE[e]
PUTRESCINE[e] <=> PUTRESCINE[c]
L-ORNITHINE[e] <=> L-ORNITHINE[c]
GLYCEROL[e] <=> GLYCEROL[c]
L-ARABINOSE[e] <=> L-ARABINOSE[c]
[c]L-ARABINOSE <=> L-RIBULOSE
[c]L-RIBULOSE + ATP -> RIBULOSE-5P + ADP
[c]RIBULOSE-5P <=> XYLULOSE-5-PHOSPHATE
D-XYLOSE[e] <=> D-XYLOSE[c]
[c]D-XYLOSE <=> XYLULOSE
[c]XYLULOSE + ATP -> XYLULOSE-5-PHOSPHATE + ADP

[1] Reactions were constrained to zero. Abbreviations: 24DINH2-PENTANOATE: 2,4-diaminopentanoate; ACET: acetate; ACETYL-P: acetylphosphate; [c]: cytoplasmic; CPD-217: D-ornithine; CPD-299: 2-amino-4-oxopentanoate; [e]: extracellular; GLT: L-glutamate; PRO: L-proline.

References

1. Schneider, J.; Eberhardt, D.; Wendisch, V.F. Improving putrescine production by *Corynebacterium glutamicum* by fine-tuning ornithine transcarbamoylase activity using a plasmid addiction system. *Appl. Microbiol. Biotechnol.* **2012**, *95*, 169–178, doi:10.1007/s00253-012-3956-9.
2. Shinfuku, Y.; Sorpitiporn, N.; Sono, M.; Furusawa, C.; Hirasawa, T.; Shimizu, H. Development and experimental verification of a genome-scale metabolic model for *Corynebacterium glutamicum*. *Microb. Cell Fact* **2009**, *8*, 43, doi:10.1186/1475-2859-8-43.

© 2015 by the authors; licensee MDPI, Basel, Switzerland. This article is an open access article distributed under the terms and conditions of the Creative Commons Attribution license (http://creativecommons.org/licenses/by/4.0/).
